# Species-specific synergistic effects of two plant growth—promoting microbes on green roof plant biomass and photosynthetic efficiency

**DOI:** 10.1371/journal.pone.0209432

**Published:** 2018-12-31

**Authors:** Long Xie, Susanna Lehvävirta, Sari Timonen, Jutta Kasurinen, Juhamatti Niemikapee, Jari P. T. Valkonen

**Affiliations:** 1 Department of Agricultural Sciences, FI, University of Helsinki, Helsinki, Finland; 2 Department of Biosciences, FI, University of Helsinki, Helsinki, Finland; 3 Department of Microbiology, FI, University of Helsinki, Helsinki, Finland; 4 Department of Bio- and Environmental Sciences, FI, University of Helsinki, Helsinki, Finland; Estacion Experimental del Zaidin, SPAIN

## Abstract

*Rhizophagus irregularis*, an arbuscular mycorrhizal fungus, and *Bacillus amyloliquefaciens*, a bacterium, are microorganisms that promote plant growth. They associate with plant roots and facilitate nutrient absorption by their hosts, increase resistance against pathogens and pests, and regulate plant growth through phytohormones. In this study, eight local plant species in Finland (*Antennaria dioica*, *Campanula rotundifolia*, *Fragaria vesca*, *Geranium sanguineum*, *Lotus corniculatus*, *Thymus serpyllum*, *Trifolium repens*, and *Viola tricolor*) were inoculated with *R*. *irregularis* and/or *B*. *amyloliquefaciens* in autoclaved substrates to evaluate the plant growth−promoting effects of different plant/microbe combinations under controlled conditions. The eight plant species were inoculated with *R*. *irregularis*, *B*. *amyloliquefaciens*, or both microbes or were not inoculated as a control. The impact of the microbes on the plants was evaluated by measuring dry shoot weight, colonization rate by the arbuscular mycorrhizal fungus, bacterial population density, and chlorophyll fluorescence using a plant phenotyping facility. Under dual inoculation conditions, *B*. *amyloliquefaciens* acted as a “mycorrhiza helper bacterium” to facilitate arbuscular mycorrhizal fungus colonization in all tested plants. In contrast, *R*. *irregularis* did not demonstrate reciprocal facilitation of the population density of *B*. *amyloliquefaciens*. Dual inoculation with *B*. *amyloliquefaciens* and *R*. *irregularis* resulted in the greatest increase in shoot weight and photosynthetic efficiency in *T*. *repens* and *F*. *vesca*.

## Introduction

Countless microorganisms reside and propagate in the rhizosphere where plant roots and soil meet. Some of the microbes have neutral or lethal effects on the growth and survival of plants, whereas others support their host plants via various mechanisms [1]. Because of their plant growth−promoting attributes, some of the microorganisms are used as commercial soil additives. *Rhizophagus irregularis* (Schenck and Smith) (formerly *Glomus intraradices*) and *Bacillus amyloliquefaciens* (Fukumoto) are among the most commonly applied plant growth−promoting microorganisms.

*R*. *irregularis* is an arbuscular mycorrhizal fungus (AMF) that is found in nearly all soil ecosystems and types [2,3]. It colonizes plants by forming intraradical hyphae and arbuscules as well as vesicles inside roots. Arbuscules are tree-like structures that function as carbohydrate/mineral/lipids exchange systems between host plants and the fungus. Vesicles are oval structures that act as nutrient reservoirs and, in some cases, as propagules [4–7]. When the arbuscules and vesicles appear, the hyphae function as nutrient transportation ducts [2]. *R*. *irregularis* facilitates plant growth by direct and indirect means, such as nutrient uptake and transportation [8,9], water absorption [10], salinity resistance [11,12], heavy-metal detoxification [13,14], and pathogen resistance [15,16].

*B*. *amyloliquefaciens* is a Gram-positive, spore-forming bacterium closely related to *Bacillus subtilis* [17]. It was given its name because it produces α-amylase and protease [18]. *B*. *amyloliquefaciens* is attracted by root exudates and lives on root surfaces. Successful colonization and effective plant growth promotion occur when a layer of bacterial cells, known as a biofilm, is formed on the surface of seeds, roots, or root hairs, as it prevents competition by other microorganisms [19–21]. Similar to *R*. *irregularis*, *B*. *amyloliquefaciens* facilitates the growth of its host plant in several ways: salt tolerance [22], drought tolerance [23], nutrient uptake [24,25], and pathogen resistance [26–28].

The use of plant growth−promoting microorganisms has increased at a rate of 10% annually in crop production worldwide over the last decade [29]. In particular, they have the potential to be used in green roofs (rooftops covered with vegetation), which are especially desirable in cities where they can provide multiple ecosystem services to urban residents, including mitigating air pollution, relieving the urban heat island effect, saving energy, and retaining stormwater [30–35]. Green roof applications are, however, limited by three major challenges: extreme weather conditions, the choice of suitable plants, and high installation and maintenance costs [36,37]. McGuire et al. [38] found that green roof soils support microbial communities that are distinct from city park soils and suggested this may be due to differences in soil depth, plant species, proximity to parks, and the conditions on the roofs. Studies have thus been conducted on green roofs to manipulate soil microbial communities [39], study AMF colonization patterns [40], and improve soil nutritional status by using AMF inoculum [41]. Nevertheless, more attention should be paid to microbe-plant interactions under green roof conditions to understand how the microbial community functions and whether microbial manipulation benefits green roof applications.

In 2012, a green roof experiment was conducted in Vantaa, Finland, led by the Fifth Dimension Green Roof Research Group. *R*. *irregularis* and *B*. *amyloliquefaciens* were added separately to the green roof plots to study microbial survival and growth. The preliminary findings suggested that the density of *B*. *amyloliquefaciens* in the rhizosphere was likely to be enhanced by *R*. *irregularis* co-inoculated in the soil. Interactions between AMF and bacteria have been reported in the literature, and these interactions can be commensalistic as well as amensalistic [42]. Mansfeld-Giese et al. [43] found that the presence of *R*. *irregularis* either promoted or suppressed the population density for 14 bacterial species. In another study using *Medicago sativa* (L.) as a host plant, co-inoculation with *Glomus deserticola* (Trappe, Bloss & Menge) and *Bacillus pumilus* (Meyer & Gottheil) increased shoot biomass and root length compared with inoculation with either of the two microbes alone [44]. Toro et al. [45] concluded that co-inoculation with *B*. *subtilis* and *R*. *irregularis* significantly increased biomass and nitrogen/phosphorous (N/P) accumulation in tissues of onion plants. If synergy between *R*. *irregularis* and *B*. *amyloliquefaciens* could be achieved in plants suitable to be grown on green roofs, it would optimize ecosystem services provided by green roofs to urban areas via plant growth enhancement.

In this study, we determined whether synergistic interactions occur between *R*. *irregularis* and *B*. *amyloliquefaciens* by setting up greenhouse experiments under controlled conditions using eight local plant species from Finland as hosts (*Antennaria dioica*, *Campanula rotundifolia*, *Fragaria vesca*, *Geranium sanguineum*, *Lotus corniculatus*, *Thymus serpyllum*, *Trifolium repens*, and *Viola tricolor*). A few of these plants, such as *T*. *serpyllum*, *T*. *repens*, and *L*. *croniculatus* [46–48], are also stress resistant and hence potentially more suitable for green roofs. Four treatments were conducted for each plant species: inoculation with (i) *R*. *irregularis*, (ii) *B*. *amyloliquefaciens*, (iii) co-inoculation with both microbes, and (iv) no microbial amendment (control). The aim was to find out which plant-microbe combinations promote the physiological performance of plants. Shoot biomass and the photosynthetic efficiency were chosen as indicators of physiological performance, as they directly reflect plant physiology and are relatively easy to measure.

## Materials and methods

### Experimentation

Experiments were conducted in the Small Plant Unit of the National Plant Phenotyping Infrastructure (NaPPI) on the Viikki campus, University of Helsinki, Finland [49] (https://www.helsinki.fi/en/infrastructures/national-plant-phenotyping). NaPPI is an in-house facility that conducts high-throughput and high-precision plant phenotyping. It consists of programmable tools for weighing, watering, and imaging the plants—both for red-green-blue pictures (RGB Camera) and chlorophyll fluorescence (Fluorecam). These tools allow comprehensive analysis of plant growth and physiology. Light-emitting diode lamps were used to avoid heating the plants by illumination.

Seed of the plant species were purchased from Suomen Niittysiemen (Jyväskylä, Finland) and stored at 4°C for 2 weeks before sowing. The inoculant products MYC4000 (4000 fungal spores of *R*. *irregularis* strain DAOM 181602 per gram) and Rhizocell (>10^9^ CFU endospores of *B*. *amyloliquefaciens* strain FZB42 per gram) were provided by Lallemand Plant Care (Castelmaurou, France). MYC4000 and Rhizocell are powdered products. They were dissolved in distilled water according to the manufacturer’s instructions before being sprayed onto the growth substrate.

Plant seeds were sown in autoclaved sand for germination. After 4 weeks, seedlings were individually transplanted into plastic pots (8 × 8 × 8 cm; VWR, Center Valley, PA, USA) filled with 450 cm^3^ autoclaved soil from a conifer forest (provided by Kekkilä Oy, Vantaa, Finland), a soil volume that is typically used for rooting plants [50–53]. The small plant unit at NaPPI is designed to fit only this particular pot size, and it is suitable for plants up to 50 cm in height. The substrates used in this study were low in nutrients, and no fertilizer was applied to keep the seedlings smaller and to more readily observe the effects of the inoculants on plant growth [52,54,55]. A shallow soil depth was used, as is typical for green roofs. The soil properties were pH 6.4; organic matter, 5.6%; soluble P, 2.2 mg/kg; and soluble N, 0.4 mg/kg. For each plant species, 24 seedlings were divided into four treatments: six seedlings were inoculated with *R*. *irregularis* (R), six seedlings with *B*. *amyloliquefaciens* (B), six seedlings were co-inoculated with both microbes (R+B), and six seedlings remained as uninoculated controls. Altogether, 192 transplanted seedlings were maintained in the NaPPI facility for 7 weeks, during which the day/night length was set to 16 h/8 h. Light intensity was 172 μmol m^−2^ s^−1^. Seedlings were irrigated daily to maintain a water-holding capacity of 65% [56]. Two independent, repeated NaPPI experiments were conducted in July 2015 and June 2016. During the first experiment, the NaPPI watering program was interrupted a few times.

Before sampling, each plant was measured for chlorophyll fluorescence activity with the Fluorecam in the NaPPI to determine the photosystem II photochemical capacity. This was calculated from the ratio of the variable fluorescence to the maximum chlorophyll fluorescence (Fv/Fm), which reflects the photosynthetic efficiency of tested plants [57]. The optimal value is 0.83 for most plant species, and a value lower than that indicates poor plant growth [58]. Plant shoots (including all aboveground biomass such as branches, leaves, and stems) were cut, desiccated at 70°C in an oven for 48 h, and weighed to measure their dry biomass. Soil adhering to roots was collected from six different places in each pot and transferred to screw cap tubes. Each soil sample was mixed by vigorous shaking and stored at 4°C. Roots were carefully brushed and stored in 70% ethanol at 4°C.

### Detection of *R*. *irregularis* in root samples

The classical AMF detection method includes root staining, microscope slide preparation, and AMF quantification [59]. After 7 weeks of cultivation in NaPPI, a root sample was collected from each of three random plants for each species and treatment. Roots were washed and stored in 70% ethanol until staining with Trypan Blue. The staining protocol was adjusted for each plant species [60] (Table 1). In brief, the roots were first soaked and softened in KOH solution, which made the staining process more effective. Then the roots were immersed in 1.5% hydrogen peroxide containing 5 ml/l ammonia (H_2_O_2_+NH_3_) to remove the background color of the cells. Next, the roots were transferred into 1% HCl solution and held in Trypan Blue solution before being stored in clear glycerol (Table 1). Finally, the roots were mounted on microscope slides in polyvinyl-lacto-glycerol solution (10 ml/l water, 10 ml/l lactic acid, 1 ml/l glycerol, and 1.66 mg/l polyvinyl alcohol).

**Table 1:** Detailed staining protocol for eight plant species.

	Staining solutions			
Plant species	KOH	H_2_O_2_+NH_3_^1^	1% HCl	Trypan Blue^2^
*C*. *rotundifolia*	60 min in 2.5% KOH at 80°C	40 min	30 min	60 min at 80°C
*L*. *corniculatus*	60 min in 2.5% KOH at 80°C	None	30 min	45 min at 95°C
*T*. *repens*	60 min in 2.5% KOH at 80°C	None	30 min	90 min at 90°C
*G*. *sanguineum*	30 min in 2.5% KOH at 121°C	None	30 min	75 min at 90°C
*F*. *vesca*	48 h in 1.25% KOH at RT^**3**^	None	60 min	60 min at 80°C
*V*. *tricolor*	60 min in 2.5% KOH at 80°C	None	30 min	75 min at 95°C
*T*. *serpyllum*	20 min in 2.5% KOH at 90°C	None	60 min	90 min at 80°C
*A*. *dioica*	48 h in 2.5% KOH at RT^3^	30 min	90 min	90 min at 90°C

^1^ 1.5% hydrogen peroxide containing 5 ml/l ammonia.

^2^ Lactic acid containing 63 ml/l glycerol, 63 ml/l water, and 0.02% Trypan Blue.

^3^ Room temperature.

A modified gridline intersect method was applied for AMF quantification [61]. There was a fine crosshair pre-scored on the ocular, and intersections of root and the vertical crosshairs were made by vertically moving the slides. When the vertical hair intersected a root, AMF structure (arbuscule, vesicle, or hypha) that was intersected by the vertical hair was recorded. Observations were categorized into eight groups: hypha (H); arbuscule (A); vesicle (V); hypha+arbuscule (HA); hypha+vesicle (HV); arbuscule+vesicle (AV); hypha+arbuscule+vesicle (HAV); and no arbuscules, vesicles, or hyphae (negative, N). A total of 100 intersections were analyzed for each slide. For instance, when calculating arbuscule rate, observations of A, HA, AV, and HAV categories were summed and divided by 100. Three plants from each species were analyzed per treatment, and data are presented as the mean ± SE.

### Detection of *B*. *amyloliquefaciens* in soil samples

The primer pair BaG3F (5´-GTCGACCACTCTTGACGTTACGGTT-3´) and BaG4R (5´-CGATCACTTCAAGATCGGCCACAG-3´), which amplifies a 94-bp fragment from *gyrB* was used to identify and quantify *B*. *amyloliquefaciens* in soil samples. Soil DNA extraction was carried out with the PowerSoil DNA Extraction Kit (MO BIO, Carlsbad, CA, USA). Genomic DNA from the Rhizocell powder was extracted with the DNeasy Plant Mini Kit (QIAGEN, Hilden, Germany). DNA concentrations were measured with a Nanodrop (Thermo Fisher, Waltham, MA, USA). PCR was carried out to amplify the target fragment from both soil DNA samples and Rhizocell DNA samples. The PCR products were sent to the Haartman Institute (Helsinki, Finland) for sequencing to verify the *Bacillus* species as the one in the Rhizocell product.

Before quantitative PCR (qPCR), soil DNA samples were diluted to 5 ng/μl, and Rhizocell DNA was diluted with Milli-Q water at ratios of 1:1, 1:10, 1:100, 1:1000, and 1:10000. The Rhizocell DNA dilutions were used to produce a standard curve and calculate amplification efficiency for each qPCR run. qPCR reactions were run with the following program: 5 min at 95°C; 45 cycles of 10 s at 95°C, 10 s at 62°C, and 10 s at 72°C; and 5 min at 72°C. *B*. *amyloliquefaciens* population densities from soil samples were calculated according to the standard curve equation [62]

$$Population\;density\;of\;Bacillus\;amyloliquefaciens=\frac{10^{(Ct-m)/-slope}\times n}{wt}$$

where “Ct” is the cycle threshold value from the qPCR; “slope” and “m” are the slope value and intercept value of the standard curve, respectively; “wt” is the weight of the soil from which the DNA was extracted; and “n” is the dilution ratio of each soil DNA sample.

### Statistical analysis

Mean values for the plant shoot dry weight, photosynthetic efficiency, and AMF colonization rate were compared using least significant difference analysis (LSD_0.05_,% confidence). Levels of significance for the treatments, host plant species, and their interactions were calculated by analysis of variance (ANOVA) using the SPSS software package (IBM SPSS Statistics 25, Armonk, NY, USA).

## Results

### *R*. *irregularis* is enhanced in R+B treatments

**Fig 1:**
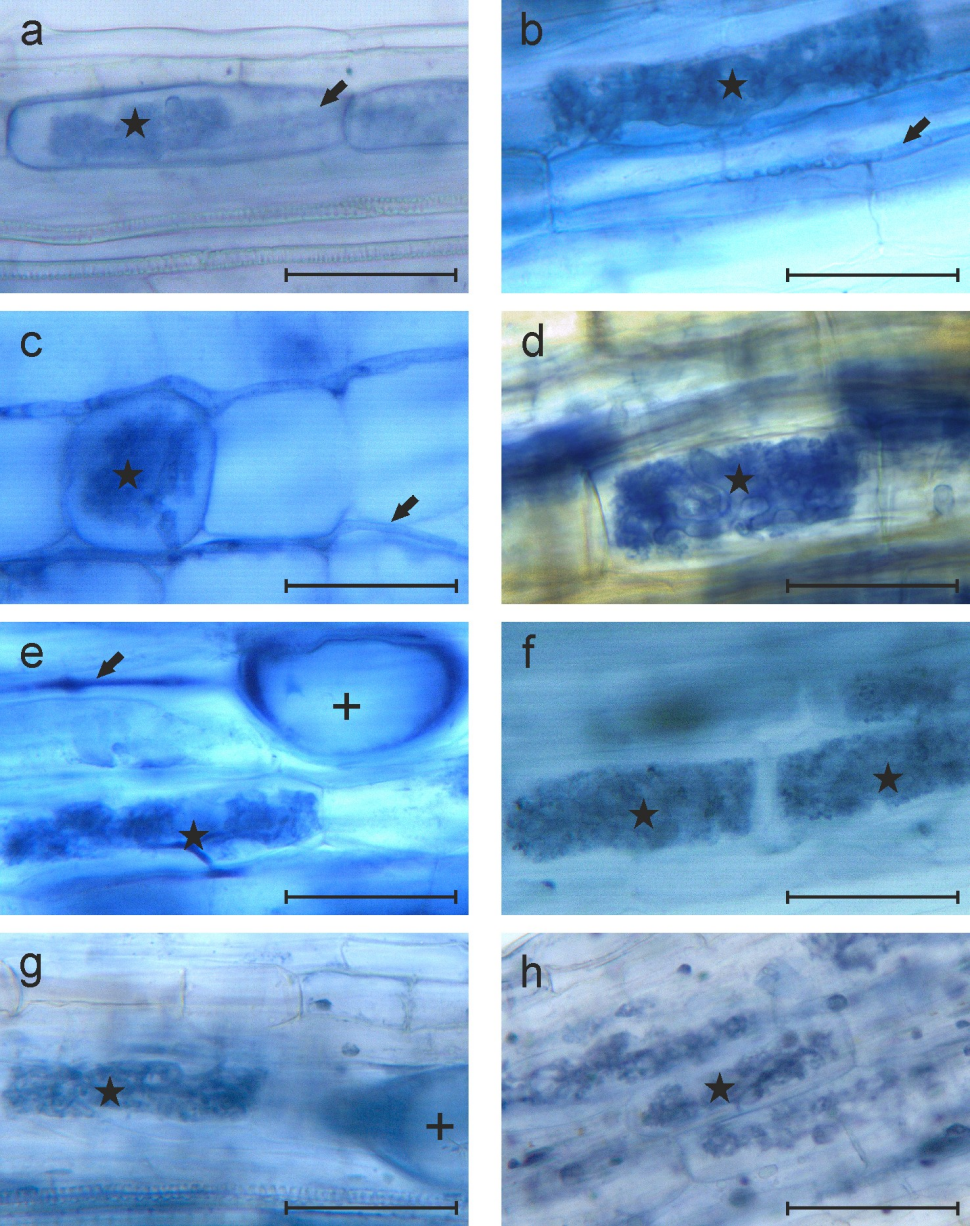
Microscopic images of AMF structures in roots of eight plants co-inoculated with *R*. *irregularis* and *B*. *amyloliquefaciens* from the second experiment. (a−h) Images from *C*. *rotundifolia* (a), *L*. *corniculatus* (b), *T*. *repens* (c), *G*. *sanguineum* (d), *F*. *vesca* (e), *V*. *tricolor* (f), *T*. *serpyllum* (g), and *A*. *dioica* (h). Scale bars represent 50 μm. A black arrow, a + symbol, and a star indicate a hypha, vesicle, and arbuscule, respectively.

Eight selected plant species were inoculated with *R*. *irregularis* or *B*. *amyloliquefaciens*, were co-inoculated with both microbes, or were untreated as controls. Two repeated NaPPI experiments were conducted to study the effect of colonization on host plants. In the first experiment, AMF structures were not detected in plants from any of the four treatments. In the second experiment, hyphae of AMF were detected in five of eight plants from treatment R, whereas arbuscules and vesicles were rare or absent in all tested plants. In the R+B treatment, AMF structures such as arbuscules and hyphae were observed in all eight plant species (Fig 1) and vesicles were observed in all tested plants except *C*. *rotundifolia*. Even though hyphae and arbuscules were detected in roots of *C*. *rotundifolia*, the colonization rate was low (3% and 1%, respectively), and no vesicles were observed (Table 2). According to the LSD_0.05_ test, colonization by the specific fungal structures was significantly higher with the R+B treatment than with R alone (P < 0.05; Table 2).

**Table 2:** Colonization by AMF of roots of eight plant species following inoculation with *R*. *irregularis* and co-inoculation with *R*. *irregularis* and *B*. *amyloliquefaciens* from the second experiment.^a^.

Plant species	Hyphae (%)		Arbuscules (%)		Vesicles (%)	
	R	R+B	R	R+B	R	R+B
*C*. *rotundifolia*	0	3.0 ± 1.0	0	1.0 ± 0.6	0	0
*L*. *corniculatus*	0	40.0 ± 3.6	0	30.7 ± 2.7	0	11.0 ± 1.5
*T*. *repens*	0	65.7 ± 2.2	0	58.0 ± 3.2	0	21.3 ± 1.2
*G*. *sanguineum*	3.0 ± 2.1	48.0 ± 2.5**	0	38.3 ± 2.6	0	8.7 ± 1.9
*F*. *vesca*	6.7 ± 3.4	95.0 ± 2.0**	2.7 ± 1.5	82.7 ± 3.2**	0	14.0 ± 2.1
*V*. *tricolor*	17.7 ± 3.2	46.0 ± 1.0**	11.7 ± 2.3	29.7 ± 4.2*	0	1.3 ± 0.9
*T*. *serpyllum*	36.7 ± 2.3	87.7 ± 1.2**	18.7 ± 0.9	47.0 ± 7.0*	2.0 ± 0.6	8.0 ± 2.0*
*A*. *dioica*	46.3 ± 1.2	82.0 ± 1.2**	13.3 ± 1.5	63.3 ± 2.3**	7.3 ± 0.7	14.0 ± 1.0**

^**a**^ Differences in the colonization rate of roots between the treatments R and R+B were tested by LSD_0.05_

*P < 0.05

**P < 0.01.

According to the AMF colonization response to co-inoculation with both microbes, the plant species could be divided into three groups: 1) plants that were not active *R*. *irregularis* host plants, regardless of the presence of *B*. *amyloliquefaciens* in the soil (*C*. *rotundifolia*); 2) plants that were *R*. *irregularis* host plants only when *B*. *amyloliquefaciens* was co-inoculated (*L*. *corniculatus* and *T*. *repens*); and 3) plants that were *R*. *irregularis* host plants when inoculated alone but became more efficiently colonized by *R*. *irregularis* when *B*. *amyloliquefaciens* was co-inoculated (*G*. *sanguineum*, *F*. *vesca*, *V*. *tricolor*, *T*. *serpyllum*, and *A*. *dioica*).

The rates of AMF structures in roots followed the pattern hyphae > arbuscules > vesicles in all the plant species, i.e., hyphae were the most prevalent AMF structure (Table 2).

### *B*. *amyloliquefaciens* colonized all tested plant species

*B*. *amyloliquefaciens* was detected in varying amounts in the soil adhering to roots in nearly all plants inoculated with the bacteria. Extraction of the DNA from the soil and sequencing of the soil DNA confirmed that the *gyrB* sequence isolated from soil DNA matched that of the bacteria in the Rhizocell product. *B*. *amyloliquefaciens* was not detected by qPCR in the soil of non-treated controls nor in soil from R treatment. According to LSD_0.05_ analysis in both experiments, no statistically significant difference (i.e., P < 0.05) in *B*. *amyloliquefaciens* population density was detected between the treatments B and R+B (Table 3).

**Table 3:** Content of *B*. *amyloliquefaciens* in the soil adhering to roots in different plant species following different treatments, as measured by qPCR.

	Plant species															
	*C*. *rotundifolia*		*L*. *corniculatus*		*T*. *repens*		*G*. *sanguineum*		*F*. *vesca*		*V*. *tricolor*		*T*. *serpyllum*		*A*. *dioica*	
Treatment	B	R+B	B	R+B	B	R+B	B	R+B	B	R+B	B	R+B	B	R+B	B	R+B
**Exp. 1** **(ng/g)**	1.5	3.6	2.0	117.0	1.7	1.6	4.8	126.4	117.4	15.5	1.5	17.8	1.7	0.5	5.8	20.4
	10.0	0	16.3	48.6	2.4	15.6	0	94.3	2.7	1.8	35.2	199.0	2.4	0.3	0.4	90
	1.4	0	1.4	3.5	0	0.5	0	0.5	2.6	0	3.2	3.2	1.7	2.8	0	41.9
	P = 0.373		P = 0.210		P = 0.408		P = 0.129		P = 0.414		P = 0.401		P = 0.430		P = 0.09	
**Exp. 2** **(ng/g)**	3.0	16.2	16.9	3.0	12.3	6.0	10.9	3.8	1.4	8.7	4.5	3.3	11.1	1.1	42.8	5.9
	0.9	1.4	7.4	6.2	19.3	3.4	1.0	5.6	2.9	3.3	2.5	1.8	16.6	1.5	1.3	0.7
	3.9	2.3	10.3	3.8	2.5	2.2	2.6	2.2	0	0.8	8.8	0	0.3	0.7	15.1	2.5
	P = 0.454		P = 0.374		P = 0.208		P = 0.779		P = 0.314		P = 0.163		P = 0.161		P = 0.246	

No significant differences were observed between the treatments B and R+B (P < 0.05; LSD_0.05_).

### Shoot weight of the plants

**Fig 2:**
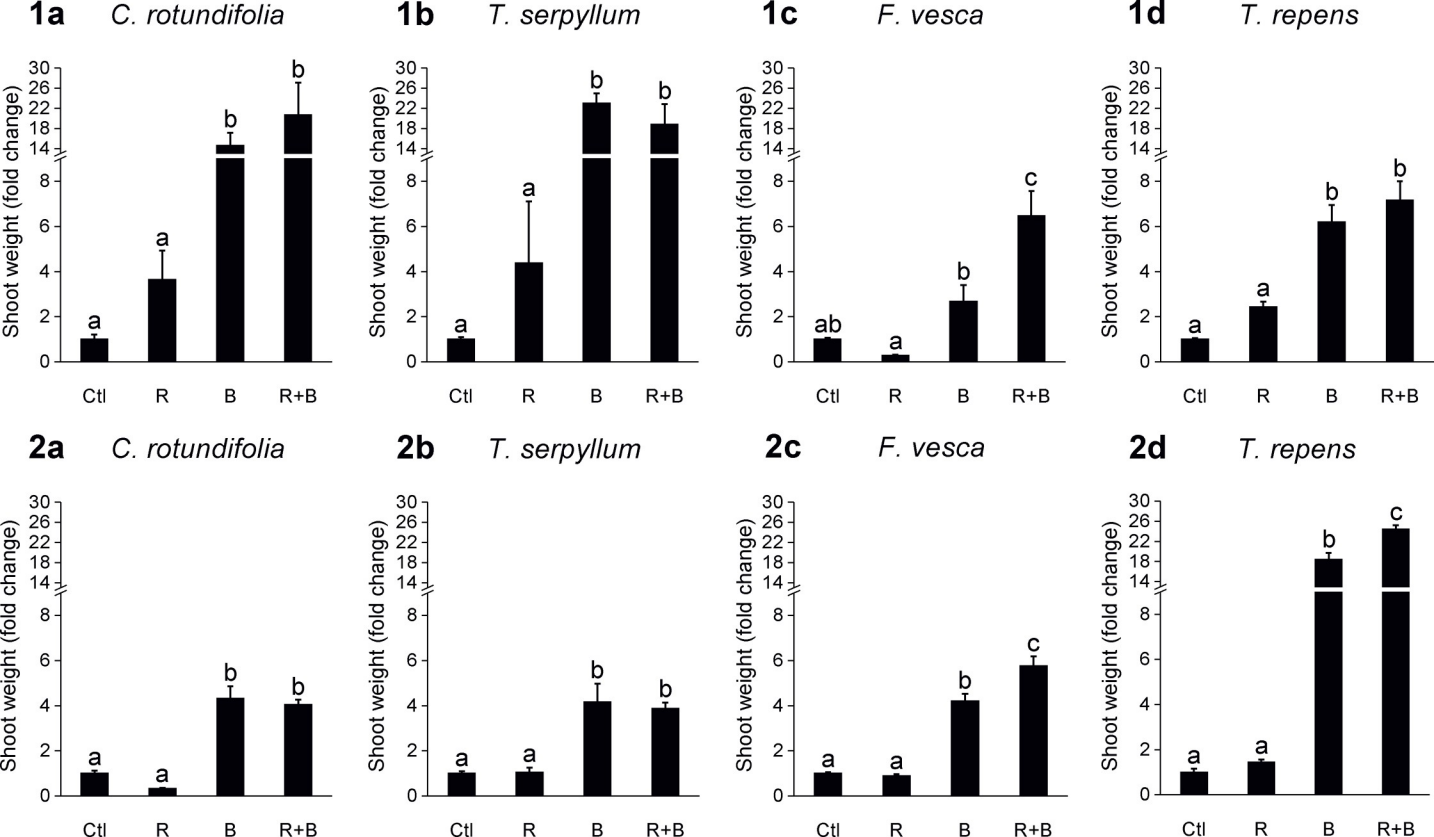
Fold change in shoot weight of four plant species whose results were consistent across the two NaPPI experiments. Bars (mean ± SE) represent fold changes in shoot weight of R, B, and R+B treated plants as compared with untreated control plants (Ctl). Graphs in the upper and lower row are from the first (1) and second (2) NaPPI experiment, respectively. (a−d) Data from *C*. *rotundifolia* (a), *T*. *serpyllum* (b), *F*. *vesca* (c), and *T*. *repens* (d). Different lowercase letters above the bars indicate statistical differences (LSD_0.05_) between the treatments at P < 0.05.

Differences in the plant shoot weight were studied in the two experiments by analyzing the fold change of the treated plants (R, B, and R+B) compared with untreated control plants. Plants were placed in two groups, namely those with consistent results (Fig 2) and those with less repeatable results (S1 Fig) obtained in the NaPPI experiments. The shoot weight of the four species with consistent results was significantly lower in untreated control plants and in R plants relative to B and R+B plants. For *C*. *rotundifolia* and *T*. *serpyllum*, no difference was detected between B and R+B, whereas for *T*. *repens* and *F*. *vesca*, plants in the R+B treatment group grew significantly larger than those in the B treatment group in at least one of the experiments.

According to an ANOVA, there was a statistically significant interaction between the host plant species and treatment for shoot weight for all plant species (S1 Table, S2 Table).

### Chlorophyll fluorescence

**Fig 3:**
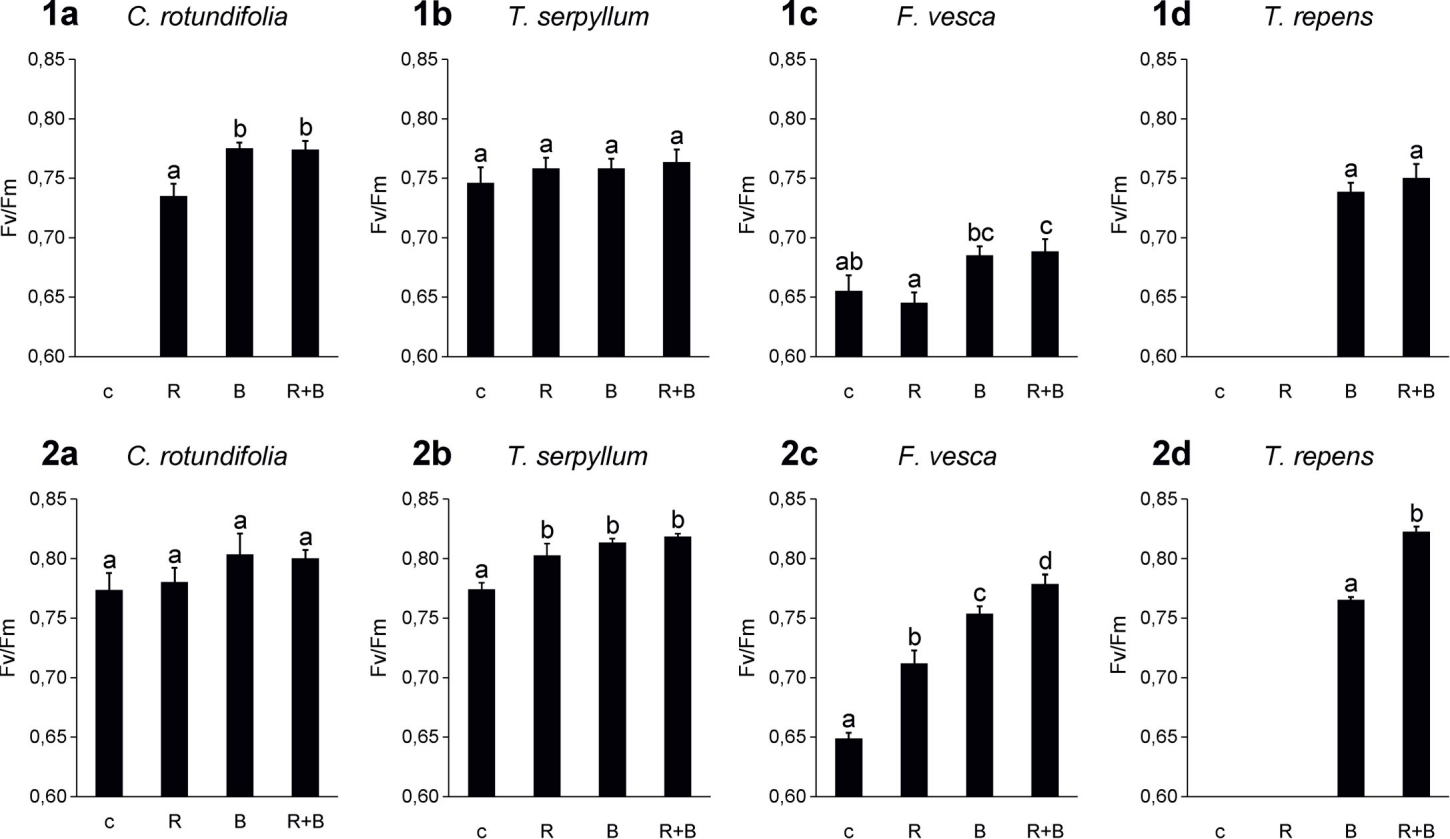
Quenching analysis of chlorophyll activities in leaves of plant species whose results were consistent in the two NaPPI experiments. Bars (mean ± SE) represent the Fv/Fm ratio of chlorophyll fluorescence for treated and untreated plants. Bar charts in the upper and lower row are from the first and second NaPPI experiment, respectively. Different lowercase letters above the bars indicate statistical differences (LSD_0.05_) between the treatments. Missing data indicate that the Fv/Fm ratio was too low to be detected.

The four plant species that showed a consistent effect for shoot weight (Fig 2) also showed reproducible patterns in photosynthetic efficiency in the two NaPPI experiments (Fig 3). For *C*. *rotundifolia* and *T*. *serpyllum*, inoculation with the microbes showed little influence on photosynthetic efficiency. For *T*. *repens* and *F*. *vesca*, the Fv/Fm ratio was lowest in untreated control plants (for *T*. *repens*, the Fv/Fm ratio in the R treatment group and in the control was too low to be detected) and was higher after B and R+B treatments. Moreover, R+B treatment in *T*. *repens* and *F*. *vesca* produced the highest Fv/Fm ratio in at least one of the experiments (Fig 3). The Fv/Fm ratios for the four plant species that showed less-consistent results in the two experiments are presented in S2 Fig and S4 Table.

The host plant species, treatment, and their interaction had significant effects on photosynthetic efficiency in all plant species (S3 Table, S4 Table).

## Discussion

Mycorrhiza helper bacterium (MHB) is a generic name for bacteria that can stimulate the formation of a mycorrhizal association with host plants [63]. In our study, the colonization rate of *R*. *irregularis* among the eight tested plant species was improved by co-inoculation with *B*. *amyloliquefaciens*. Moreover, for some plant species (*L*. *corniculatus* and *T*. *repens*), successful colonization by *R*. *irregularis* could only happen when *B*. *amyloliquefaciens* was co-inoculated. We can infer that AMF colonization in some plant species was strongly dependent on the presence of a MHB (*B*. *amyloliquefaciens* in this case).

Our findings are consistent with the studies of Yusran et al. [64], which indicated that different species of MHB promote mycorrhization to different levels and that co-inoculation with a MHB and an AMF has a positive impact on biomass and phytopathogen control in tomato plants. Our results show that the colonization-promoting effects of *B*. *amyloliquefaciens* on *R*. *irregularis* can occur across a broad range of host plants suggesting that such a promoting effect is fungus specific, rather than host plant specific [65,66]. Many MHB species have been identified, and it is predicted that more are likely to be discovered [44,67]. Finding such beneficial combinations would be promising for sustainable and cost-effective urban greening, and potential combinations should be extensively tested for their reliability and consistency under field conditions before wider exploitation.

The exact processes and compounds of microbes that are involved in stimulation of AMF colonization by MHB remain mostly unknown [68,69]. It does not necessarily require physical attachment but could happen through chemical signaling [63]. One possible mechanism behind these effects could be the release of gaseous volatiles, such as 2-methyl isoburneol, geosmin, and CO_2_, by the MHB that stimulates the growth of AMF. In addition to gaseous chemicals, other active metabolites produced by a MHB include vitamins, amino acids, and growth substances, any or all of which might directly stimulate the growth of an AMF [63,70].

The present study indicated that co-inoculation with *R*. *irregularis* and *B*. *amyloliquefaciens* led to increased plant biomass of at least *T*. *repens* and *F*. *vesca* in repeated experiments. A similar synergistic effect has been reported in a study that combined four *Glomus* species, namely *G*. *aggregatum* (Schenck and Smith), *G*. *fasciculatum* (Gerd & Trappe), *G*. *intraradices*, and *G*. *mosseae* (Nicolson & Gerd), with *B*. *subtilis* on *Pelargonium graveolens* (L'Her. ex Aiton). All four combinations (each of the four *Glomus* species inoculated separately with *B*. *subtilis*) resulted in synergistic effects to various degrees, as measured by increases in the plant dry weight of 25.7% to 74.3% as compared with that of control plants and of 7.8% to 14.5% as compared with inoculation with a single *Glomus* species [71].

We found that AMF colonization at a low level may not have any promoting effects on host plants. It is especially true if the internal hyphae are the only AMF structure present in the roots. However, little scientific data are available concerning the threshold level of arbuscules and vesicles needed for a significant promoting effect, and further studies on this topic are needed. *C*. *rotundifolia* hardly differed in biomass production between the two treatments R and R+B. A possible explanation could be that *C*. *rotundifolia* is highly tolerant to drought, soil pH, environmental variations, frost, and strong wind, so that it does not need symbiosis for further growth-promoting effects [72]. In contrast, Nuortila et al. [73] reported that, instead of increasing plant biomass, AMF colonization can significantly decrease the plant biomass of *C*. *rotundifolia* by 33% and can reduce flowers by 66%. Furthermore, in some specific AMF−host plant combinations, AMF can act as a “hitchhiker” that profits from the mycorrhizal-host network without returning benefits back to the host [74,75]. In the present study, *T*. *serpyllum* reached high levels of AMF colonization after the R+B treatment in the second experiment, but no significant difference in biomass production between R+B and B plants was observed. Similar results have been obtained using *Thymus vulgaris* (L.) as a host plant and *G*. *mosseae* as the AMF inoculant: a higher AMF colonization rate after AMF treatment did not increase plant biomass [76]. The phenomenon depends on the plant-AMF combinations, water availability, and soil types [77].

Enhanced photosynthetic efficiency is another benefit that AMF and plant growth−promoting rhizobacteria (PGPR) provide to some host plants, such as *T*. *repens* and *F*. *vesca* in the present study. A single inoculation with AMF or PGPR may significantly promote photosynthetic efficiency under suboptimal conditions such as high salinity and nutrient deficiency in the soil. For example, co-colonization by *Glomus* and *Bacillus* species enhances the photosynthetic efficiency of lettuce plants, compared with colonization by either of the two microbes. The extent of the synergistic effect is greatly dependent on the *Glomus* and *Bacillus* species combination [70].

Quantification of *B*. *amyloliquefaciens* revealed varying population densities between replicates in our study. Trevors et al. [78] also found large variation between replicates in their study of PGPR in the soil. It appears challenging to correlate PGPR population density with effects that promote plant growth [79], but the information would be useful, as beyond a certain PGPR population density no further enhancement may occur [79–82]. This scenario also seems possible in our study, as higher population densities of *B*. *amyloliquefaciens* did not lead to a higher biomass production or photosynthetic efficiency.

Our results suggest that neither a positive nor negative interaction was found between AMF colonization rate and the abundance of *B*. *amyloliquefaciens* in the soil in any of the eight tested plant species. These findings are supported by Kostoula et al. [83], who got similar results with *Crithmum maritimum* (L.) as a host plant and *G*. *intraradices* and *B*. *amyloliquefaciens* as inoculants. They found no significant effect of *G*. *intraradices* colonization on the population density of *B*. *amyloliquefaciens* in the soil. Likewise, Alam et al. [71] found no effect of four *Glomus* species on *B*. *subtilis* population density.

Interruptions in the NaPPI system during the first experiment made growth conditions unstable, which probably explains why the shoot weight and photosynthetic efficiency in half of the plant species were not reproducible across the two NaPPI experiments. Still, the other four plant species exhibited relatively consistent patterns in shoot weight and photosynthetic efficiency, suggesting that the plant species studied show different sensitivities to the stability of their growth environment.

In conclusion, for host plants *T*. *repens* and *F*. *vesca*, *B*. *amyloliquefaciens* could act as a MHB to facilitate colonization by *R*. *irregularis*, and dual inoculation with *B*. *amyloliquefaciens* and *R*. *irregularis* should result in higher shoot weight and photosynthetic efficiency. Such mycorrhiza-helping effects and dual-inoculation growth-promoting effects in other test plants were more conditional and dependent on the environment. Further field experiments to test these effects of co-infection on green roofs should focus on *T*. *repens* and *F*. *vesca*, as they showed consistent and promising results, and they are likely to produce better growth than other plants under such conditions.

## Supporting information

S1 Table Effect of microbial colonization and host species on shoot dry weight of four plant species whose results were consistent in the two NaPPI experiments. (DOC)

S2 Table Effect of microbial colonization and host species on shoot dry weight of four plant species whose results were inconsistent in the two NaPPI experiments. (DOC)

S3 Table Effect of microbial colonization and host species on the photosynthetic efficiency of four plant species whose results were consistent in the two NaPPI experiments. (DOC)

S4 Table Effect of microbial colonization and host species on photosynthetic efficiency of four plants whose results were inconsistent in the two NaPPI experiments. (DOC)

S1 Fig Fold changes in shoot weight of four plant species whose results were inconsistent in the two NaPPI experiments. (TIF)

S2 Fig Quenching analysis of chlorophyll acivities in leaves of four plant species whose results were inconsistent in the two NaPPI experiments. (TIF)
