# Supplementary material for: Species-specific synergistic effects of two plant growth—promoting microbes on green roof plant biomass and photosynthetic efficiency
Source: PLoS One. 2018 Dec 31;13(12):e0209432. doi: 10.1371/journal.pone.0209432 (PMC6312232; doi:10.1371/journal.pone.0209432)
Supplement: S1 Table — (DOC) [file pone.0209432.s001.doc]

**S1 Table. Effect of microbial colonization and host species on shoot dry weight of four plant species whose results were consistent in the two NaPPI experiments.**

| **Plant species** | **Experiment** | **Dry biomass, mg (SE) ^a^** | | | |
| --- | --- | --- | --- | --- | --- |
|  |  | Control | R | B | R+B |
| *C. rotundifolia* | 1 | 0.28 (0.06) | 1.02 (0.36) | 4.10 (0.71) | 5.80 (1.79) |
|  | 2 | 3.23 (0.39) | 1.08 (0.09) | 14.03 (1.66) | 13.13 (0.64) |
| *T. repens* | 1 | 1.30 (0.07) | 3.17 (0.29) | 8.06 (0.98) | 9.32 (2.16) |
|  | 2 | 4.83 (0.76) | 7.00 (0.58) | 89.13 (6.21) | 118.2 (3.51) |
| *F. vesca* | 1 | 4.52 (0.33) | 1.27 (0.18) | 12.15 (3.24) | 29.27 (4.95) |
|  | 2 | 6.08 (0.29) | 5.42 (0.45) | 25.73 (1.77) | 35.72 (2.46) |
| *T. serpyllum* | 1 | 1.13 (0.1) | 4.96 (3.07) | 26.02 (2.22) | 21.28 (4.51) |
|  | 2 | 5.30 (0.49) | 5.55 (1.12) | 22.15 (4.26) | 20.59 (1.36) |

^a^ P-values (ANOVA): Plant species <0.001, Treatment <0.001, and Species × Treatment <0.001.
