# Supplementary material for: Species-specific synergistic effects of two plant growth—promoting microbes on green roof plant biomass and photosynthetic efficiency
Source: PLoS One. 2018 Dec 31;13(12):e0209432. doi: 10.1371/journal.pone.0209432 (PMC6312232; doi:10.1371/journal.pone.0209432)
Supplement: S2 Table — (DOC) [file pone.0209432.s002.doc]

**S2 Table**. Effect of microbial colonization and host species on shoot dry weight of four plant species whose results were inconsistent in the two NaPPI experiments.

| Plant species | Experiment | Dry biomass, mg (SE) **^a^** | | | |
| --- | --- | --- | --- | --- | --- |
|  |  | Control | R | B | R+B |
| *L. corniculatus* | 1 | 5.10 (0.07) | 3.87 (0.45) | 13.55 (2.44) | 9.30 (1.09) |
|  | 2 | 6.55 (0.44) | 8.62 (1.29) | 16.12 (0.87) | 131.23 (5.81) |
| *G. sanguineum* | 1 | 45.14 (3.36) | 54.10 (5.56) | 61.50 (5.45) | 45.20 (4.07) |
|  | 2 | 59.45 (2.06) | 54.83 (4.53) | 90.74 (3.78) | 163.38 (12.32) |
| *V. tricolor* | 1 | 2.33 (0.14) | 3.30 (0.10) | 25.10 (4.39) | 13.50 (1.11) |
|  | 2 | 5.93 (0.23) | 7.77 (0.65) | 16.28 (1.88) | 42.22 (1.97) |
| *A. dioica* | 1 | 1.18 (0.05) | no data | 5.00 (1.60) | 6.46 (1.32) |
|  | 2 | 3.32 (0.21) | 5.52 (0.47) | 5.45 (1.21) | 20.90 (0.84) |

^a^ P-values (ANOVA): Plant species <0.001, Treatment <0.001, and Species × Treatment <0.05.
