# Supplementary material for: Species-specific synergistic effects of two plant growth—promoting microbes on green roof plant biomass and photosynthetic efficiency
Source: PLoS One. 2018 Dec 31;13(12):e0209432. doi: 10.1371/journal.pone.0209432 (PMC6312232; doi:10.1371/journal.pone.0209432)
Supplement: S3 Table — (DOC) [file pone.0209432.s003.doc]

**S3 Table. Effect of microbial colonization and host species on the photosynthetic efficiency of four plant species** **whose results were consistent in the two NaPPI experiments. ^a^**

| **Plant species** | **Experiment** | **Chlorophyll fluorescence, Fv/Fm (SE**) **^b^** | | | |
| --- | --- | --- | --- | --- | --- |
|  |  | Control | R | B | R+B |
| *C. rotundifolia* | 1 | no data | 0.735 (0.010) | 0.775 (0.005) | 0.774 (0.007) |
|  | 2 | 0.773 (0.015) | 0.780 (0.012) | 0.803 (0.018) | 0.800 (0.007) |
| *T. repens* | 1 | no data | no data | 0.738 (0.008) | 0.750 (0.012) |
|  | 2 | no data | no data | 0.765 (0.003) | 0.823 (0.004) |
| *F. vesca* | 1 | 0.655 (0.013) | 0.645 (0.009) | 0.705 (0.005) | 0.688 (0.010) |
|  | 2 | 0.648 (0.005) | 0.712 (0.011) | 0.798 (0.011) | 0.778 (0.008) |
| *T. serpyllum* | 1 | 0.763 (0.011) | 0.758 (0.009) | 0.746 (0.013) | 0.758 (0.009) |
|  | 2 | 0.774 (0.006) | 0.803 (0.010) | 0.813 (0.003) | 0.819 (0.003) |

^a^‘No data’ indicates that leaves of the plant were too small for the Fluorecam to pick up any signal.

^b^ P-values (ANOVA): Plant species <0.001, Treatment <0.001, and Species × Treatment <0.01.
