# Supplementary material for: Species-specific synergistic effects of two plant growth—promoting microbes on green roof plant biomass and photosynthetic efficiency
Source: PLoS One. 2018 Dec 31;13(12):e0209432. doi: 10.1371/journal.pone.0209432 (PMC6312232; doi:10.1371/journal.pone.0209432)
Supplement: S4 Table — (DOC) [file pone.0209432.s004.doc]

**S4 Table. Effect of microbial colonization and host species on photosynthetic efficiency of four plants whose results were inconsistent in the two NaPPI experiments.**

| **Plant species** | **Experiment** | **Chlorophyll fluorescence, Fv/Fm (SE**) **^a^** | | | |
| --- | --- | --- | --- | --- | --- |
|  |  | Control | R | B | R+B |
| *L. corniculatus* | 1 | 0.740 (0.011) | 0.765 (0.007) | 0.763 (0.007) | 0.758 (0.010) |
|  | 2 | 0.762 (0.005) | 0.774 (0.011) | 0.773 (0.007) | 0.800 (0.004) |
| *G. sanguineum* | 1 | 0.789 (0.004) | 0.787 (0.013) | 0.780 (0.000) | 0.782 (0.013) |
|  | 2 | 0.772 (0.007) | 0.795 (0.004) | 0.796 (0.007) | 0.820 (0.006) |
| *V. tricolor* | 1 | 0.637 (0.033) | 0.620 (0.090) | 0.713 (0.018) | 0.695 (0.007) |
|  | 2 | 0.682 (0.018) | 0.752 (0.018) | 0.758 (0.010) | 0.780 (0.008) |
| *A. dioica* | 1 | no data | no data | 0.685 (0.008) | 0.715 (0.013) |
|  | 2 | 0.748 (0.016) | 0.792 (0.012) | 0.753 (0.007) | 0.824 (0.002) |

^a^ P-values (ANOVA): Plant species <0.001, Treatment <0.001, and Species × Treatment <0.05.
