# Supplementary figures and images for: Species-specific synergistic effects of two plant growth—promoting microbes on green roof plant biomass and photosynthetic efficiency
Source: PLoS One. 2018 Dec 31;13(12):e0209432. doi: 10.1371/journal.pone.0209432 (PMC6312232; doi:10.1371/journal.pone.0209432)

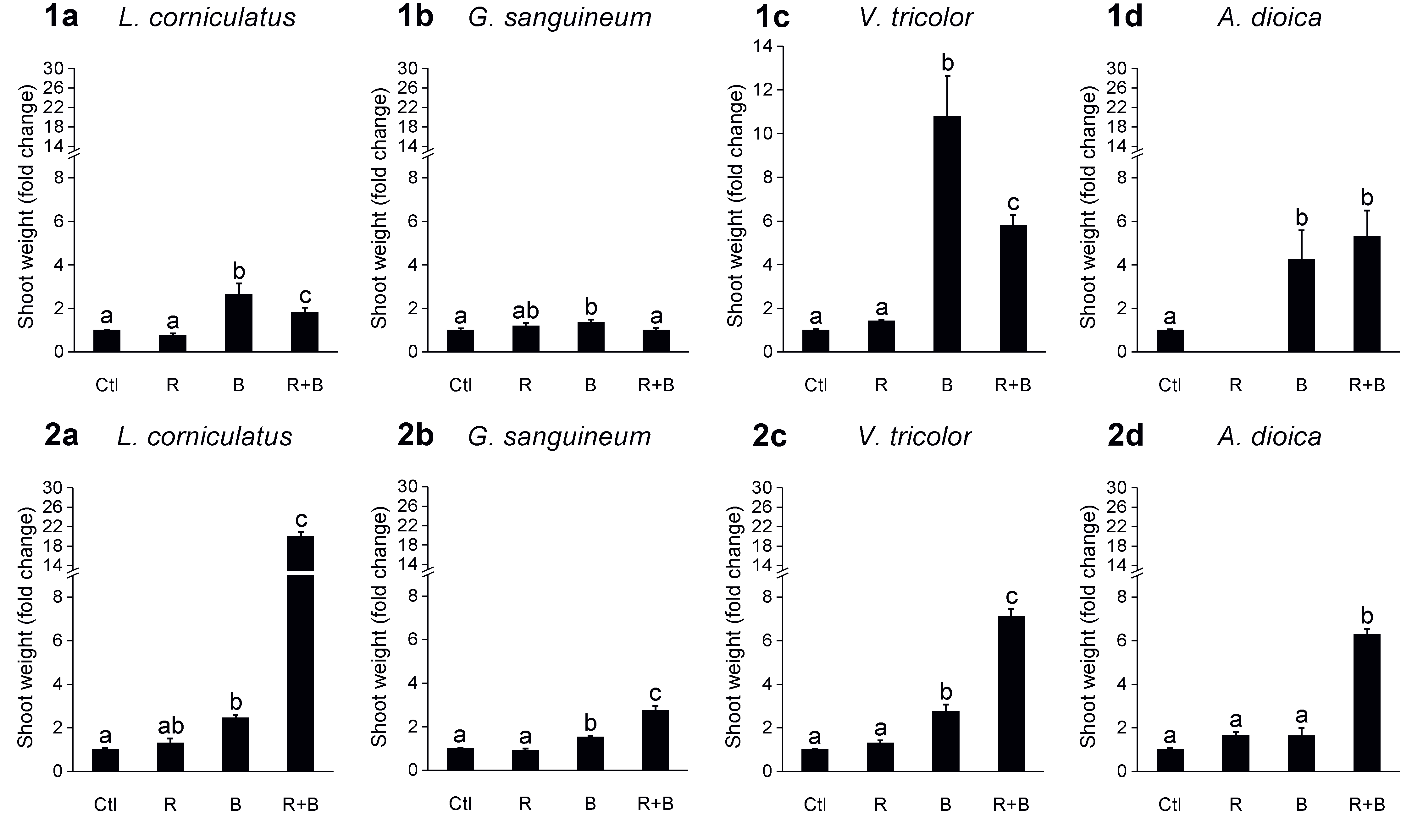

Supplement: S1 Fig — (TIF) [file pone.0209432.s005.tif]

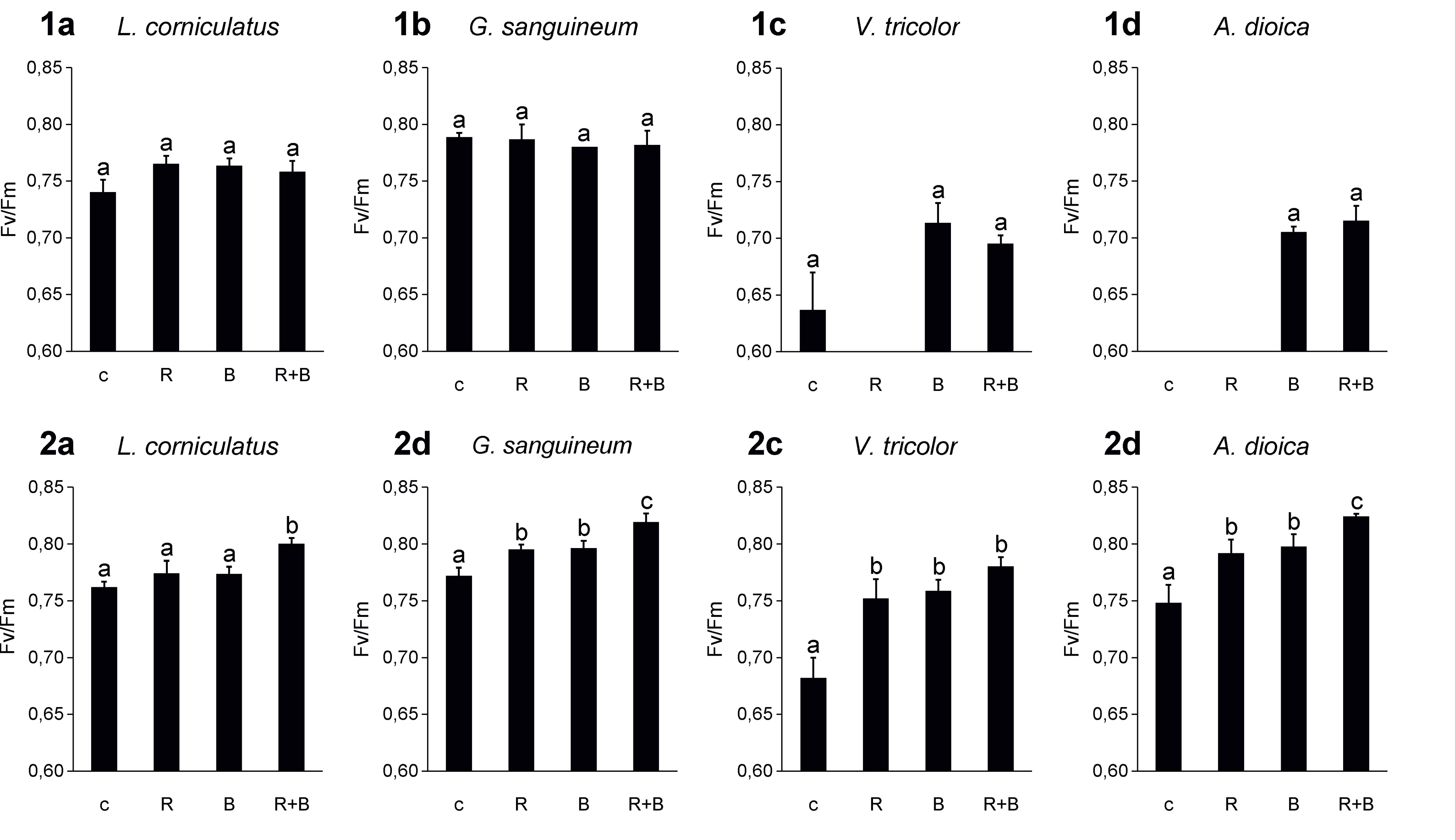

Supplement: S2 Fig — (TIF) [file pone.0209432.s006.tif]
